# Supplementary material for: Investigating the role of group-based morality in extreme behavioral expressions of prejudice
Source: Nat Commun. 2021 Jul 28;12:4585. doi: 10.1038/s41467-021-24786-2 (PMC8319297; doi:10.1038/s41467-021-24786-2)
Supplement: Supplementary file 1 — Supplementary information [file 41467_2021_24786_MOESM1_ESM.pdf]

Supplemental Information

Investigating the role of group-based morality in extreme behavioral expressions of prejudice

Joe Hoover[1,2], Mohammad Atari[1,2], Aida Mostafazadeh Davani[2,3,*], Brendan Kennedy[2,3,*], Gwenyth Portillo-Wightman[1], Leigh Yeh[3]

Morteza Dehghani[1,2,3]

[1]Department of Psychology, University of Southern California, CA, USA

[2]Brain and Creativity Institute, University of Southern California, CA, USA

[3]Department of Computer Science, University of Southern California, CA, USA

Supplemental Information

Investigating the role of group-based morality in extreme behavioral expressions of prejudice

Study 1

To characterize the demographic composition of the YourMorals sample in Study 1, Supplementary Table 1 shows the proportion of the sample in particular age ranges and education levels. The largest demographic group in the YourMorals sample was between the ages of 20–29 and with a Bachelor's degree or some college.

Supplementary Table 1

YourMorals age and education sample characteristics

Age	Education	N	Sample_Proportion
20-29	Bachelor's degree or some college	25451	0.233
20-29	Graduate degree	16249	0.149
20-29	HS grad or less	1192	0.011
30-44	Bachelor's degree or some college	13486	0.123
30-44	Graduate degree	16465	0.151
30-44	HS grad or less	647	0.006
45-64	Bachelor's degree or some college	11454	0.105
45-64	Graduate degree	18457	0.169
45-64	HS grad or less	649	0.006
Over 65	Bachelor's degree or some college	1499	0.014
Over 65	Graduate degree	3636	0.033
Over 65	HS grad or less	140	0.001

Next, in order to validate the estimated county-level proportion of conservatives produced by the MrsP model in Study 1 against a ground truth measure, Supplementary Figure 1 plots county-level 2016 share of Republican votes by estimates of county-level proportion. The correlation ($r = 0.63$, 95% CI = [0.60, 0.65]) between the estimated measure and the observed, ground truth measure of ideological distribution at the county-level further suggests the validity of the county-level estimates produced by MrsP. Supplementary Figure 2 compares the association between

predicted and actual Republican share visually.

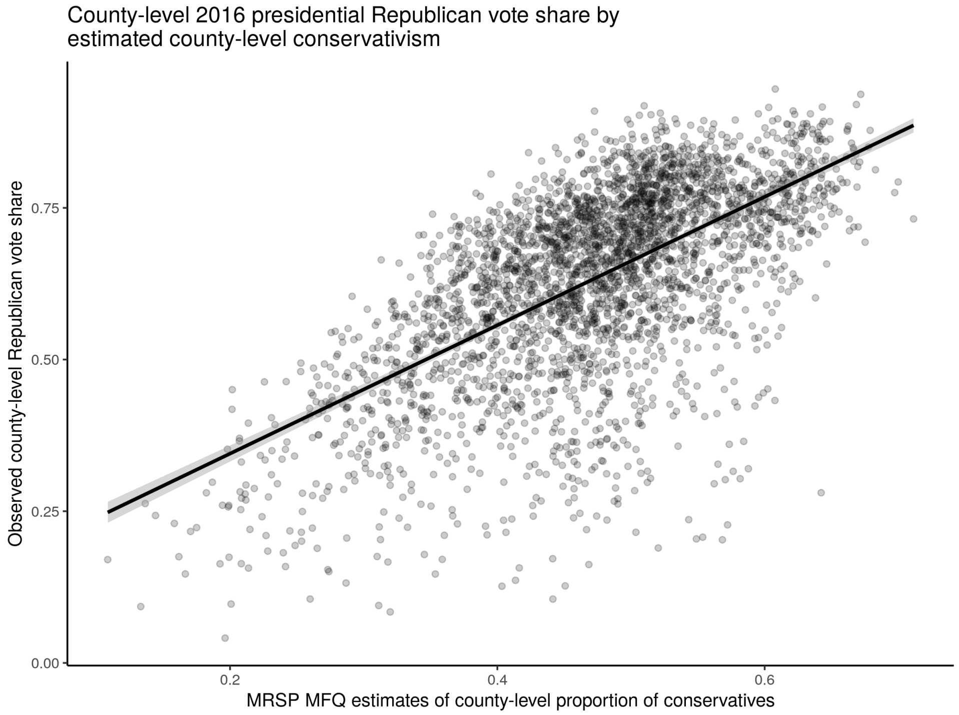

Supplementary Figure 1. Observed county-level 2016 Republican Presidential vote share by estimated county-level proportion of conservative residents

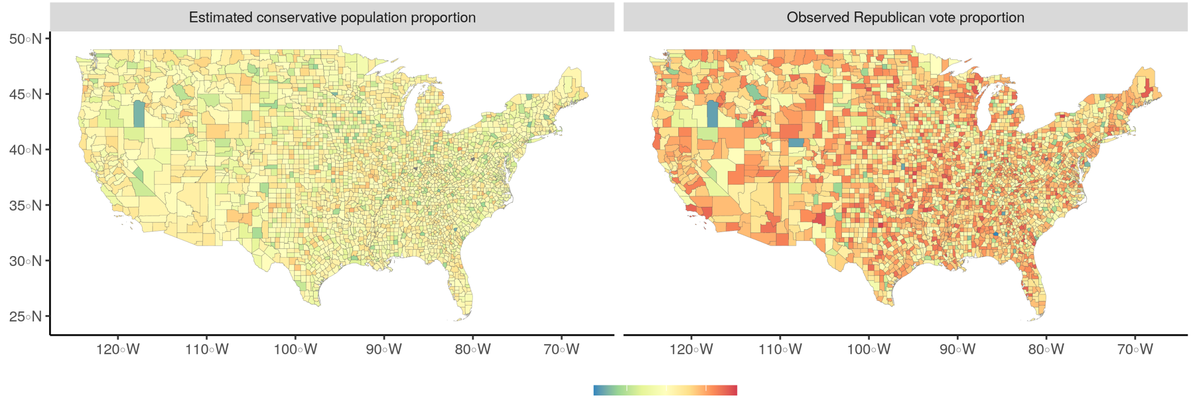

Supplementary Figure 2. Estimated county-level proportion of Conservatives (Left) and observed county-level 2016 Republican Presidential vote share (Right)

Supplementary Table 2

Models of the county-level rate of hate groups with (Model 2) and without (Model 1) state fixed effects

	Model 1	Model 2
Intercept	−2.264*** (0.082)	−1.693*** (0.307)
Individualizing Std.	−0.087 (0.098)	−0.436*** (0.146)
Binding Std.	0.320*** (0.102)	0.190 (0.183)
% with 4 year college degree Std.	0.332*** (0.078)	0.251*** (0.085)
% non-Hispanic White Std.	−0.091 (0.086)	−0.069 (0.104)
% Below poverty line Std.	0.204*** (0.073)	0.207** (0.082)
2016 Presidential Vote Share Std.	0.010 (0.150)	0.125 (0.229)
Rural	0.376*** (0.114)	0.222* (0.121)
AR		−0.352 (0.407)
AZ		0.083 (0.685)
CA		−0.500 (0.472)
CO		−0.589 (0.517)
CT		−2.390** (1.106)
DC		2.089 (1.922)
DE		−1.508 (1.277)
FL		−0.795* (0.407)
GA		−0.797** (0.354)
IA		−1.881*** (0.552)
ID		−0.033 (0.515)
IL		−0.900** (0.454)
IN		0.100 (0.424)
KS		−1.605*** (0.535)
KY		−0.894** (0.400)
LA		−0.355 (0.407)
MA		−0.997 (0.817)
MD		−0.536 (0.553)
ME		−0.844 (0.843)
MI		−0.638 (0.443)
MN		−1.815*** (0.516)

MO		−0.531 (0.406)
MS		0.035 (0.392)
MT		−0.210 (0.530)
NC		−1.080*** (0.384)
ND		−1.119* (0.658)
NE		−0.598 (0.482)
NH		0.877 (0.831)
NJ		0.655 (0.577)
NM		−0.030 (0.578)
NV		−0.334 (0.762)
NY		−0.371 (0.524)
OH		−0.930** (0.437)
OK		−1.098** (0.442)
OR		−1.102* (0.644)
PA		0.145 (0.480)
RI		−2.314 (1.627)
SC		−0.797* (0.467)
SD		−0.652 (0.540)
TN		−0.055 (0.375)
TX		−0.040 (0.330)
UT		−1.614** (0.720)
VA		0.333 (0.362)
VT		0.559 (0.836)
WA		−0.720 (0.573)
WI		−0.697 (0.476)
WV		−0.821* (0.475)
WY		−34.406 (5,181,562.000)
Observations	3,017	3,017
Log Likelihood	−2,664.876	−2,598.414
Akaike Inf. Crit.	5,345.751	5,308.827

Note: *p<0.1; **p<0.05; ***p<0.01

Study 2

Here we report model estimates for each of the three regression models estimated for Study 2 (See main text for discussion). Specifically, we report mean posterior estimates with Highest Posterior Density Intervals for the fixed and random effects estimated via these models (See Supplementary Table 3). We also report estimates from models that adjust for individual-level political ideology (M = 2.97, SD = 2.01; See Supplementary Table 4). Note, for these models, N = 508 as 3 participants indicated that they either did not know or have a political ideology.

Mediation Analysis

To investigate whether perceived moral wrongdoing statistically mediated the effect of the Binding values, we relied on Bayesian posterior simulation to estimate average mediation effects and average direct effects[1;2;3;4]. We used this framework because standard approaches to mediation analysis are not appropriate for ordered logistic regression outcome models[1] due to their fixed error variance[3] and because it offers a holistic approach to mediation estimation for hierarchical models[5].

To evaluate the effects of standardized Binding values, we focus on expected changes in PMW and EBEP justification given a change from 0 to 1 in standardized Binding values (i.e. the difference between being at the mean of Binding values vs being one standard deviation above the mean).

Method. Under this approach, the average mediation effect (AME; e.g. indirect effect) for a mediator M is estimated via posterior simulations from two Bayesian regression models. In the first of these models, M is regressed on the independent or treatment variable T. In the second of these models, the endogenous dependent variable Y is regressed on both M and T.

Next, the model posteriors are used to generate a set of counterfactual predictions that are used to estimate the AME and average direct effect (ADE). Specifically, the first model is used to simulate N predicted values of $M|T = T$. For example, this might involve simulating two sets of values for M by drawing 500 values

for $M|T = \textit{control}$ and 500 values for $M|T = \textit{experimental}$ from the model posterior. Per convention, we represent these sets of simulated values as $M_{T=0}$ and $M_{T=1}$, respectively, where $M_{T=0}$ represents the conditional posterior distribution of M when treatment equals zero.

Then, $M_{T=0}$ and $M_{T=1}$, the simulated values generated in the previous step, are used to simulate the expected values of $Y|T = t, M = m$. Specifically, three sets of values are simulated for Y: $Y|T = 0, M = M_{T=1}$, $Y|T = 1, M = M_{T=0}$, $Y|T = 0, M = M_{T=0}$. These simulation sets approximate the conditional distribution of Y given treatment equals t and the M equals a plausible value under a given treatment condition. For example, the first set, which we represent as $Y_{T=0,M_{T=1}}$, approximates the expected distribution of Y where $T = 0$ but M is set as if $T = 1$. That is, $Y_{T=0,M_{T=1}}$ estimates the posterior distribution of Y for values of M expected under the treatment condition while setting T to zero. Similarly, the second and third sets, $Y_{T=1,M_{T=0}}$ and $Y_{T=0,M_{T=0}}$, approximate the posterior distributions for Y when $T = 1$ but M is set as if $T = 0$ and for Y when $T = 0$ and M is set as if $T = 0$. Thus, in contrast to $Y_{T=0,M_{T=1}}$, $Y_{T=1,M_{T=0}}$ estimates the posterior distribution of Y under the treatment condition while effectively blocking the effect of M and $Y_{T=0,M_{T=0}}$ estimates the posterior distribution of Y under the control condition.

Finally, the simulation sets $Y_{T=0,M_{T=1}}$, $Y_{T=1,M_{T=0}}$, and $Y_{T=0,M_{T=0}}$ are used to estimate the AME and ADE. Specifically, the posterior distribution of the AME is calculated as $Y_{T=0,M_{T=1}} - Y_{T=0,M_{T=0}}$ and the ADE is calculated as $Y_{T=1,M_{T=0}} - Y_{T=0,M_{T=0}}$. Accordingly, the AME estimates the counterfactual change in Y that is expected given that the treatment is held constant at control but the mediator is changed as if the treatment was administered. Similarly, the ADE estimates the counterfactual change in Y that is expected given that the treatment is administered, but the mediator is restricted to values consistent with the control condition.

Because this approach to mediation estimation relies on model predictions, concerns about coefficient comparisons across models are irrelevant. Importantly, even for generalized linear models, it also enables direct estimates of mediation effects on the

scale of the dependent variable. Thus, combining this approach with ordered logistic regression allows us to estimate AMEs and ADEs for the probabilities of selecting each response level of the dependent variable. Finally, this approach also facilitates estimating AMEs and ADEs while adjusting for covariates. To do this, covariates are included in the regression models and then conditioned on at specific levels during the simulation step.

Using this approach, we estimated AMEs and ADEs for perceived moral wrongdoing (M), experimental condition (T) and EBEP justification (Y) using the two-step process outlined above. Further, we estimated these effects both without and with adjustments for political ideology.

First, for both the low and high moral threat conditions, we used Model 1 to simulate two sets of 500 perceived moral wrongdoing scores, which we represent as $M_{T=0}$ and $M_{T=1}$, respectively. Then, for each EBEP item j, we used Model 3 to simulate three sets, each consisting of 500 draws, of EBEP justification scores conditional on experimental condition and the simulated moral wrongness scores, $Y^j_{T=0,M_{T=1}}$, $Y^j_{T=1,M_{T=0}}$, and $Y^j_{T=0,M_{T=0}}$. For all posterior draws from Model 3, we conditioned on the random effects of EBEP item and marginalized over the random effects of subject. Thus, for each of the four EBEP items, this process yielded posterior approximations of $Y^j_{T=0,M_{T=1}}$, $Y^j_{T=1,M_{T=0}}$, and $Y^j_{T=0,M_{T=0}}$, such that each simulation set consisted of 500 (simulated moral wrongness scores) $\times 500$ (posterior draws) $= 250,000$ values.

Finally, we calculated AMEs and ADEs for each $j \in j = 1, ..., 4$ EBEP items as well as marginal AME and ADE across all EBEP items as

$$AME^j = \frac{1}{N_j}(\sum_{i=1}^{i=N_j} Y_{ij,T=0,M_{T=1}} - Y_{ij,T=0,M_{T=0}})$$
$$ADE^j = \frac{1}{N_j}(\sum_{i=1}^{i=N_j} Y_{ij,T=1,M_{T=0}} - Y_{ij,T=0,M_{T=0}})$$
$$AME = \frac{1}{\sum_{j=1}^{j=4} N_j}(\sum_{j=1}^{j=4}\sum_{i=1}^{i=N} Y_{ij,T=0,M_{T=1}} - Y_{ij,T=0,M_{T=0}})$$
$$ADE = \frac{1}{\sum_{j=1}^{j=4} N_j}(\sum_{j=1}^{j=4}\sum_{i=1}^{i=N} Y_{ij,T=1,M_{T=0}} - Y_{ij,T=0,M_{T=0}}),$$

where $N = 250{,}000$.

Mediation Results

As noted above, our mediation procedure yielded AME and ADE estimates for the probability of selecting a given EBEP response level for each EBEP item and a given EBEP response level marginalized across EBEP items. Here, we present all of these estimates, as well as summary estimates that represent the AMEs and ADEs for responses ≥ "slightly justified".

Study Materials for Study 2

EBEP items

> Now, we would like you to answer some questions about potential behaviors toward Muslims in the U.S..
>
> Before answering each of these questions, please imagine an average American. We'll call him Dave.
>
> Please imagine that Dave is angry and upset because he believes Muslims are hurting his community.
>
> 1. Now, imagine that Dave makes a Facebook post. In his post, he writes that "Muslims are a virus infecting our community" and that "the infection must be stopped."
>
> 2. Please imagine Dave, an average American who is angry and upset because he believes Muslims are hurting his community.
>
> Imagine that Dave distributes flyers in his neighborhood. On the flyers, he draws Muslims as viruses and writes, "the infection must be stopped."
>
> 3. Please imagine Dave, an average American who is angry and upset because he believes Muslims are hurting his community.
>
> Imagine that Dave yells at a member of the Muslim community, shouting slurs and telling them to "get out of my neighborhood."

4. Please imagine Dave, an average American who is angry and upset because he believes Muslims are hurting his community.

Imagine that Dave mutters a slur and pushes a member of the Muslim community, starting a fight. Imagine that Dave punches them several times.

Study 3

Here we report model estimates for each of the three regression models estimated for Study 3 (See main text for discussion). Specifically, we report mean posterior estimates with Highest Posterior Density Intervals for the fixed and random effects estimated via these models (See Supplementary Table 9). We also report estimates from models that adjust for individual-level political ideology ($M = 2.28$, $SD = 1.66$; See Supplementary Table 10). Note, for these models, $N = 313$ as 11 participants indicated that they either did not know or have a political ideology.

Study Materials for Study 3

Vignette

Webster Springs, Ill. — Over the last year, Webster Springs has changed dramatically due to the increasing population of Mexican immigrants.

Rising rates of poverty and crime have caused tension between long-time residents and the new community of immigrants, many of whom have entered the country without documentation.

“They come from a place where they’re used to not having very much,” local mill-worker Dave Johnston said. “They work for wages that can’t support an American family.”

According to Johnston and other native residents, many community members have had to leave their jobs and collect unemployment in order to provide for their families.

These vacant positions have been quickly taken by Mexican immigrants, allowing employers to avoid paying the wages that native workers need.

Despite residents concerns, the Mexican community has continued growing at a rapid rate and it will soon form a large minority in Webster Springs.

EBEP items

Now, we would like you to answer some questions about potential reactions to Mexican immigration in Webster Springs.

Before answering each of these questions, please imagine a native resident of Webster Springs. We'll call him Dave.

1. Please imagine that Dave is unable to find work.

Now, imagine that Dave posts the following message on Facebook:

"Slowly, the majority of Americans are waking up to the fact that they are hated in their own country. 'Diversity' wasn't a failed experiment, it was a disguised invasion meant to replace America's founding stock. The Mexican invasion is just the latest wave in this war. Whatever the cost, THEY MUST BE STOPPED!!!"

2. Please imagine Dave, a native resident of Webster Springs who is unable to find work.

Imagine that he distributes fliers in Webster Springs with the following message:

"Slowly, the majority of Americans are waking up to the fact that they are hated in their own country. 'Diversity' wasn't a failed experiment, it was a disguised invasion meant to replace America's founding stock. The Mexican invasion is just the latest wave in this war. Whatever the cost, THEY MUST BE STOPPED!!!"

3. Please imagine Dave, a native resident of Webster Springs who is unable to find work.

Imagine that Dave yells at a member of the Mexican community, shouting slurs and telling them to "get out of Webster Springs."

4. Please imagine Dave, a native resident of Webster Springs who is unable to find work.

Imagine that Dave punches a member of the Mexican community several times, shouting slurs at them and breaking their nose.

Debriefing Statement

Thank you participating in our survey!

Before you leave, we want you to know that the news story that you read was fabricated. Webster Springs Illinois does not exist.

The purpose of this study was to better understand how people feel about issues related to immigration depending on whether they believe immigrants do or do not pose a threat.

Mediation Results

To investigate whether perceived moral wrongdoing statistically mediated the effect of the Binding values, we used the same approach as in Study 2. Specifically, we estimated AMEs and ADEs for PMW (M) using standardized Binding values as the exogenous treatment variable and perceived EBEP justification as the outcome variable. To evaluate the effects of standardized Binding values, we focus on expected changes in PMW and EBEP justification given a change from 0 to 1 in standardized Binding values (i.e. the difference between being at the mean of Binding values vs being one standard deviation above the mean). All procedural steps were otherwise identical to those used for the mediation analysis reported for Study 2.

Study 4

Regression Models

Here we report model estimates for each of the three regression models estimated for Study 4 (See main text for discussion). Specifically, we report mean posterior estimates with Highest Posterior Density Intervals for the fixed and random effects

estimated via these models (See Supplementary Table 15). We also report estimates from models that adjust for individual-level political ideology (M = 2.67, SD = 1.78; See Supplementary Table 16). Note, for these models, N = 278 as 10 participants indicated that they either did not know or have a political ideology.

Mediation Results

To investigate whether perceived moral wrongdoing statistically mediated the effect of the Binding values, we used the same approach as in Study 2. Specifically, we estimated AMEs and ADEs for PMW (M) using standardized Binding values as the exogenous treatment variable and perceived EBEP justification as the outcome variable. To evaluate the effects of standardized Binding values, we focus on expected changes in PMW and EBEP justification given a change from 0 to 1 in standardized Binding values (i.e. the difference between being at the mean of Binding values vs being one standard deviation above the mean). All procedural steps were otherwise identical to those used for the mediation analysis reported for Study 2.

Study Materials for Study 3

Vignettes

High threat condition

> Webster Springs, Ill. — Over the last year, Webster Springs has changed dramatically due to the increasing population of Sandirian immigrants.
>
> Rising rates of poverty and crime have caused tension between long-time residents and the new community of immigrants, many of whom have entered the country without documentation.
>
> “They come from a place where they’re used to not having very much,” local mill-worker Dave Johnston said. “They work for wages that can’t support an American family.”

According to Johnston and other native residents, many community members have had to leave their jobs and collect unemployment in order to provide for their families.

These vacant positions have been quickly taken by new Sandirian immigrants, allowing employers to avoid paying the wages that native workers need.

Despite residents concerns, the Sandirian community has continued growing at a rapid rate and it will soon form a large minority in Webster Springs.

Low threat condition

Webster Springs, Ill. — Over the last year, little has changed in Webster Springs despite an increasing population of Sandirian immigrants.

Webster Springs' economy has absorbed the new workforce and relations between long-term residents and the new immigrant community have remained largely amicable.

"They come from a place where they're used to not having very much," local mill-worker Dave Johnston said. "They work hard and go home, just like the rest of us."

According to Johnston and other long-time residents, the growing presence of Sandirian has had little effect on their daily lives, aside from helping local businesses fill jobs that have long been vacant.

While the Sandirian population has increased, the community is still a small minority in Webster Springs.

Debriefing statement

Thank you participating in our survey!

Before you leave, we want you to know that the news story that you read was fabricated. Sandirians are not a real group and Webster Springs Illinois does not exist.

> The purpose of this study was to better understand how people feel about issues related to immigration depending on whether they believe immigrants do or do not pose a threat.

Study 5

To better understand the differences between the moderated ACMEs in the Binding and Individualizing conditions, we focus on two key sets of parameters (See Supplementary Tables 21 and 24 for all model estimates). First, we examine the degree to which the relevant moral values measure (e.g., Binding values or Individualizing values) moderated the effect of moral threat on perceived moral wrongness. These parameters indicate whether people high on Binding (Individualizing) values were more likely to see the social outgroup's behavior as immoral in the Binding (Individualizing) condition. If one of these parameters was not distinguishable from zero, that would suggest that the manipulation of moral threat did not reliably target the relevant moral domain. Importantly, in both conditions, our models indicate that people higher on the relevant moral domain were more likely to see the social outgroup's behavior as immoral, $\beta_{Binding} = 0.30$, 95% CI = [0.16, 0.43], $\beta_{Individualizing} = 0.30$, 95% CI = [0.17, 0.43].

Next, we examine the degree to which the relevant moral values moderated the effect of perceived moral wrongness on EBEP justification. Similar to the first set of parameters, these parameters reflect the degree to which the effect of perceived moral wrongness on EBEP justification varies depending on people's moral values. Importantly, in both conditions, we found that PME had a strong, positive effect on EBEP justification, as expected under the Moralized Threat Hypothesis, $\beta_{Binding} =$ 2.43, 95% CI = [1.84, 2.95] and $\beta_{Individualizing} = 2.38$, 95% CI = [0.10, -0.35, 0.54]. However, while we also found that Binding values moderate the effect of PME on EBEPs in the Binding values condition, $\beta = 0.62$, 95% CI = [0.06, 1.23], Individualizing values do not moderate the effect of PME on EBEPs in the Individualizing values condition, $\beta = 0.10$, 95% CI = [-0.35, 0.54].

Study Materials for Study 5

Vignettes

Control condition vignette:

Webster Springs, FL. — Some members of a 1200-member community known as the People of the Earth have been raising produce in their community garden outside Webster Springs, Florida, according to a recent report in the Webster Springs Journal.

Commenting on the report, Webster Springs Mayor Lori Gladwell said, "Some of the People of the Earth have been raising a variety of fruits and vegetables."

"They are raising tomatoes, squash, lettuce and potatoes" she said.

Members of the community have pointed out that not every member contributes to the community garden, but they believe it is an important part of their community. "For many of us, raising and sharing our own produce is a wonderful practice," said the People of the Earth's founder Joseph Brownwell.

Since the report was published, Webster Springs residents have not expressed much interest in the People of the Earth's community garden.

Binding Values Condition Vignette:

Webster Springs, FL. — Some disciples of a 1200-member community known as the People of the Earth have started engaging in sexual rituals involving faeces, semen, and menstrual blood, according to a recent Webster Springs Journal interview with an ex-member of the community.

Commenting on the report, Webster Springs Mayor Lori Gladwell said, "We now know that some of the People of the Earth have been engaging in extreme sexual rituals."

"These people are eating each other's diarrhea and drinking fermented semen and menstrual blood. It's disgusting," she said.

However, it is not clear how officials can address the issue because what the People of the Earth refer to as "sex magic" is not prohibited by Florida law.

Members of the community are quick to point out that not every member practices "sex magic", but they believe it is a sacred tenet of their faith. "For many of us, consuming and sharing the sacred fruit of our bodies is a wonderful ritual for seeking our lord," said People of the Earth Elder Joseph Brownwell.

Nonetheless, some Webster Springs residents are outraged.

"What these people are doing is wrong. Drinking rotten semen and menstrual blood...It's immoral and a sin against nature," local delivery driver Dave Johnston said. "Somebody needs to do something about it."

Individualizing Values Condition Vignette:

Webster Springs, FL. — Some members of a 1200-member community known as the People of the Earth have started raising dogs and cats for human consumption, according to a recent Webster Springs Journal interview with an ex-member of the community.

Commenting on the report, Webster Springs Mayor Lori Gladwell said, "We now know that some of the People of the Earth have been using dogs and cats for food."

"These people are slaughtering and cooking their own dogs and cats, animals that many people see as loving companions. It's tragic and inhumane," she said.

However, it is not clear how officials can address the issue because what the People of the Earth refer to as "pet recycling" is not prohibited by Florida law.

Members of the community are quick to point out that not every member practices "pet recycling", but they believe that it is an important tenet of their approach to sustainability. "For many of us, pets like dogs and cats are a wonderful source of food," said People of the Earth Elder Joseph Brownwell.

Nonetheless, some Webster Springs residents are outraged.

"What these people are doing is wrong. Slaughtering little animals that don't do anything but love us...It's immoral and inhumane," local delivery driver Dave Johnston said. "Somebody needs to do something about it."

Debriefing statement

Thank you participating in our survey!

Before you leave, we want you to know that the news story that you read was fabricated. The People of the Earth are not a real group and Webster Springs does not exist.

The purpose of this study was to better understand how people feel about issues related to out-group behavior depending on whether they believe the out-group has done something immoral.

Supplementary Table 3

Study 2 Model Estimates

Fixed Effects		Model 1<br>PMW Std.	Model 2<br>EBEP	Model 3<br>EBEP
	Intercept	0.00		
		[−0.08; 0.08]		
	I Values Std.	−0.25*	−1.70*	−1.29*
		[−0.34; −0.17]	[−2.33; −1.11]	[−1.86; −0.66]
	B Values Std.	0.44*	2.29*	1.48*
		[0.35; 0.52]	[1.65; 2.94]	[0.81; 2.16]
	PMW Std.			1.72*
				[0.68; 2.67]
	Intercept[1]		1.40	1.57
			[−2.18; 4.31]	[−1.62; 4.10]
	Intercept[2]		3.26	3.44*
			[−0.46; 6.09]	[0.27; 6.00]
	Intercept[3]		4.21*	4.42*
			[0.61; 7.18]	[1.32; 7.06]
	Intercept[4]		5.80*	6.07*
			[2.06; 8.64]	[2.90; 8.65]
	Intercept[5]		6.86*	7.16*
			[3.25; 9.82]	[3.96; 9.77]
	Intercept[6]		8.62*	8.97*
			[4.87; 11.58]	[5.88; 11.74]
SD of Random Effects				
	$\text{Intercept}_{Subject}$		4.41*	4.16*
			[3.90; 4.96]	[3.66; 4.65]
	Intercept_{EBEP}		3.00*	2.51*
			[0.84; 6.36]	[0.76; 5.39]
	$\text{I Values Std}_{EBEP}$		0.27*	0.31*
			[0.00; 0.77]	[0.00; 0.89]
	$\text{B Values Std}_{EBEP}$		0.20*	0.27*
			[0.00; 0.63]	[0.00; 0.84]
	PMW Std_{EBEP}			0.70*
				[0.10; 1.77]

* 0 outside 95% highest posterior density interval

Supplementary Table 4

Study 2 Model Estimates Adjusted for Ideology

Fixed Effects		Model 1	Model 2	Model 3
		PMW Std.	EBEP	EBEP
	Intercept	0.00		
		[−0.07; 0.07]		
	I Values Std.	−0.09*	−1.22*	−1.08*
		[−0.17; −0.00]	[−1.95; −0.43]	[−1.85; −0.29]
	B Values Std.	0.25*	1.70*	1.31*
		[0.16; 0.34]	[1.00; 2.50]	[0.58; 2.09]
	PMW Std.			1.48*
				[0.60; 2.44]
	Ideology Std.	0.41*	1.29*	0.67
		[0.33; 0.50]	[0.31; 2.20]	[−0.14; 1.43]
	Intercept[1]		1.61	1.62
			[−1.75; 4.18]	[−1.43; 4.36]
	Intercept[2]		3.49*	3.51*
			[0.30; 6.26]	[0.40; 6.20]
	Intercept[3]		4.47*	4.50*
			[1.12; 7.11]	[1.27; 7.11]
	Intercept[4]		6.11*	6.17*
			[2.81; 8.79]	[2.90; 8.74]
	Intercept[5]		7.19*	7.27*
			[3.90; 9.88]	[4.07; 9.96]
	Intercept[6]		8.96*	9.08*
			[5.73; 11.77]	[5.99; 11.92]
SD of Random Effects				
	$\text{Intercept}_{Subject}$		4.38*	4.19*
			[3.85; 4.91]	[3.70; 4.70]
	Intercept_{EBEP}		2.65*	2.52*
			[0.79; 5.63]	[0.70; 5.32]
	I Values Std_{EBEP}		0.43*	0.43*
			[0.00; 1.15]	[0.00; 1.22]
	B Values Std_{EBEP}		0.31*	0.36*
			[0.00; 0.96]	[0.00; 1.08]
	PMW Std_{EBEP}			0.59*
				[0.00; 1.61]
	Ideology Std_{EBEP}		0.63*	0.48*
			[0.09; 1.62]	[0.00; 1.27]

* 0 outside 95% highest posterior density interval

Supplementary Table 5

Study 2 Mediation Estimates for PMW for EBEP ≥ "Slightly justified"

EBEP	AME	ADE	Total
Facebook	**0.01** [0, 0.07]	**0.02** [0, 0.13]	**0.03** [0, 0.2]
Flyer	**0.01** [0, 0.07]	**0.02** [0, 0.15]	**0.03** [0, 0.25]
Yell	**0.01** [0, 0.05]	**0.02** [0, 0.15]	**0.03** [0, 0.22]
Assault	**0.01** [0, 0.06]	**0.02** [0, 0.13]	**0.03** [0, 0.21]
Marginal	**0.01** [0, 0.06]	**0.02** [0, 0.15]	**0.03** [0, 0.23]

Supplementary Table 6

Study 2 Mediation Estimates for PMW for EBEP ≥ "Slightly justified" Adjusted for Ideology

EBEP	AME	ADE	Total
Facebook	**0.003** [0, 0.026]	**0.018** [0, 0.159]	**0.021** [0.001, 0.174]
Flyer	**0.003** [0, 0.029]	**0.017** [0, 0.137]	**0.02** [0.001, 0.161]
Yell	**0.003** [0, 0.02]	**0.015** [0, 0.122]	**0.018** [0, 0.146]
Assault	**0.003** [0, 0.015]	**0.014** [0, 0.115]	**0.016** [0, 0.126]
Marginal	**0.003** [0, 0.02]	**0.015** [0, 0.123]	**0.018** [0, 0.147]

Supplementary Table 7

Study 2 Mediation Estimates for PMW at Each Response Level

EBEP	Response Level	AME	ADE	Total
Facebook	1	**-0.112** [-0.26, -0.01]	**-0.246** [-0.45, -0.04]	**-0.358** [-0.64, -0.05]
	2	0.066 [-0.09, 0.16]	0.133 [-0.18, 0.27]	0.2 [-0.27, 0.38]
	3	**0.022** [0, 0.08]	**0.051** [0, 0.15]	**0.073** [0, 0.2]
	4	**0.017** [0, 0.1]	**0.042** [0, 0.19]	**0.059** [0, 0.27]
	5	**0.004** [0, 0.03]	**0.01** [0, 0.07]	**0.014** [0, 0.11]
	6	**0.002** [0, 0.02]	**0.006** [0, 0.04]	**0.008** [0, 0.06]
	7	**0.001** [0, 0]	**0.003** [0, 0.01]	**0.005** [0, 0.01]
Flyer	1	**-0.116** [-0.25, -0.01]	**-0.252** [-0.44, -0.04]	**-0.368** [-0.63, -0.08]
	2	0.064 [-0.12, 0.16]	0.133 [-0.19, 0.27]	0.198 [-0.29, 0.39]
	3	0.023 [0, 0.08]	0.052 [-0.01, 0.13]	**0.075** [0, 0.2]
	4	**0.02** [0, 0.11]	**0.045** [0, 0.21]	**0.065** [0, 0.3]
	5	**0.005** [0, 0.04]	**0.012** [0, 0.09]	**0.017** [0, 0.14]
	6	**0.003** [0, 0.02]	**0.007** [0, 0.05]	**0.01** [0, 0.08]
	7	**0.001** [0, 0.01]	**0.002** [0, 0.01]	**0.003** [0, 0.02]
Yell	1	**-0.111** [-0.26, -0.01]	**-0.241** [-0.45, -0.04]	**-0.353** [-0.62, -0.06]
	2	0.064 [-0.08, 0.16]	0.131 [-0.19, 0.28]	0.195 [-0.27, 0.39]
	3	**0.022** [0, 0.08]	**0.049** [0, 0.13]	**0.071** [0, 0.2]
	4	**0.018** [0, 0.1]	**0.042** [0, 0.19]	**0.06** [0, 0.28]
	5	**0.005** [0, 0.03]	**0.011** [0, 0.08]	**0.016** [0, 0.13]
	6	**0.002** [0, 0.01]	**0.007** [0, 0.05]	**0.009** [0, 0.06]
	7	**0** [0, 0]	**0.002** [0, 0.01]	**0.002** [0, 0.01]
Assault	1	**-0.115** [-0.25, -0.01]	**-0.252** [-0.46, -0.04]	**-0.366** [-0.63, -0.07]
	2	0.067 [-0.1, 0.15]	0.138 [-0.16, 0.27]	0.205 [-0.26, 0.38]
	3	**0.022** [0, 0.08]	0.051 [0, 0.14]	**0.073** [0, 0.2]
	4	**0.018** [0, 0.1]	**0.043** [0, 0.2]	**0.061** [0, 0.28]
	5	**0.004** [0, 0.04]	**0.01** [0, 0.07]	**0.015** [0, 0.11]
	6	**0.002** [0, 0.02]	**0.006** [0, 0.04]	**0.009** [0, 0.07]
	7	**0.001** [0, 0]	**0.002** [0, 0.01]	**0.003** [0, 0.02]

Supplementary Table 8

Study 2 Mediation Estimates for PMW at Each Response Level Adjusted for Ideology

EBEP	Response Level	AME	ADE	Total
Facebook	1	**-0.051** [-0.13, 0]	**-0.209** [-0.42, -0.02]	**-0.259** [-0.49, -0.03]
	2	0.032 [-0.04, 0.08]	0.117 [-0.16, 0.27]	0.149 [-0.21, 0.31]
	3	0.009 [0, 0.04]	0.041 [-0.03, 0.13]	0.05 [-0.01, 0.16]
	4	**0.007** [0, 0.04]	**0.033** [0, 0.17]	**0.04** [0, 0.21]
	5	**0.002** [0, 0.01]	**0.009** [0, 0.08]	**0.011** [0, 0.09]
	6	**0.001** [0, 0.01]	**0.006** [0, 0.06]	**0.007** [0, 0.06]
	7	**0** [0, 0]	**0.002** [0, 0.01]	**0.003** [0, 0.02]
Flyer	1	**-0.051** [-0.13, 0]	**-0.206** [-0.41, -0.03]	**-0.258** [-0.5, -0.04]
	2	0.031 [-0.04, 0.08]	0.116 [-0.18, 0.25]	0.147 [-0.2, 0.3]
	3	0.009 [0, 0.04]	0.04 [-0.02, 0.13]	0.049 [-0.01, 0.16]
	4	**0.007** [0, 0.05]	**0.034** [0, 0.18]	**0.042** [0, 0.23]
	5	**0.002** [0, 0.02]	**0.009** [0, 0.08]	**0.011** [0, 0.1]
	6	**0.001** [0, 0.01]	**0.005** [0, 0.04]	**0.006** [0, 0.05]
	7	**0** [0, 0]	**0.002** [0, 0.01]	**0.002** [0, 0.01]
Yell	1	**-0.051** [-0.13, 0]	**-0.212** [-0.43, -0.03]	**-0.263** [-0.53, -0.04]
	2	0.031 [-0.04, 0.08]	0.115 [-0.18, 0.27]	0.145 [-0.21, 0.31]
	3	**0.01** [0, 0.04]	**0.044** [0, 0.14]	**0.054** [0, 0.16]
	4	**0.008** [0, 0.05]	**0.038** [0, 0.19]	**0.046** [0, 0.23]
	5	**0.002** [0, 0.01]	**0.009** [0, 0.07]	**0.011** [0, 0.09]
	6	**0.001** [0, 0.01]	**0.005** [0, 0.04]	**0.006** [0, 0.05]
	7	**0** [0, 0]	**0.001** [0, 0.01]	**0.001** [0, 0.01]
Assault	1	**-0.051** [-0.12, 0]	**-0.208** [-0.41, -0.02]	**-0.26** [-0.5, -0.03]
	2	0.034 [-0.02, 0.08]	0.123 [-0.15, 0.26]	0.156 [-0.15, 0.31]
	3	**0.009** [0, 0.04]	**0.041** [0, 0.13]	**0.05** [0, 0.15]
	4	**0.006** [0, 0.04]	**0.031** [0, 0.17]	**0.037** [0, 0.2]
	5	**0.001** [0, 0.01]	**0.007** [0, 0.06]	**0.008** [0, 0.07]
	6	**0.001** [0, 0]	**0.005** [0, 0.03]	**0.006** [0, 0.04]
	7	**0** [0, 0]	**0.002** [0, 0.01]	**0.002** [0, 0.01]

Supplementary Table 9

Study 3 Model Estimates

Fixed Effects		Model 1	Model 2	Model 3
		PMW Std.	EBEP	EBEP
	Intercept	0.00		
		[−0.09; 0.09]		
	I Values Std.	−0.16*	−1.15*	−0.87*
		[−0.26; −0.06]	[−1.91; −0.40]	[−1.70; −0.03]
	B Values Std.	0.47*	1.60*	0.73*
		[0.37; 0.56]	[1.10; 2.11]	[0.21; 1.19]
	PMW Std.			1.63*
				[0.74; 2.51]
	Intercept[1]		0.20	0.19
			[−3.89; 3.93]	[−3.69; 3.98]
	Intercept[2]		2.34	2.35
			[−1.82; 6.03]	[−1.79; 5.89]
	Intercept[3]		3.45	3.47
			[−0.59; 7.26]	[−0.73; 6.96]
	Intercept[4]		4.70*	4.73*
			[0.60; 8.51]	[0.54; 8.22]
	Intercept[5]		6.42*	6.51*
			[2.16; 10.12]	[2.40; 10.13]
	Intercept[6]		8.13*	8.28*
			[3.95; 11.96]	[4.17; 11.97]
SD of Random Effects				
	$\text{Intercept}_{Subject}$		2.83*	2.39*
			[2.43; 3.24]	[2.01; 2.75]
	Intercept_{EBEP}		4.12*	3.94*
			[1.43; 8.01]	[1.41; 7.78]
	$\text{I Values Std}_{EBEP}$		0.57*	0.63*
			[0.03; 1.45]	[0.01; 1.61]
	$\text{B Values Std}_{EBEP}$		0.21*	0.23*
			[0.00; 0.69]	[0.00; 0.75]
	PMW Std_{EBEP}			0.61*
				[0.00; 1.70]

* 0 outside 95% highest posterior density interval. Estimates for Models 2 and 3 reported on log scale.

Supplementary Table 10

Study 3 Model Estimates Adjusted for Ideology

Fixed Effects		Model 1	Model 2	Model 3
		PMW Std.	EBEP	EBEP
	Intercept	−0.01		
		[−0.10; 0.09]		
	I Values Std.	−0.07	−0.68	−0.59
		[−0.18; 0.04]	[−1.61; 0.33]	[−1.55; 0.39]
	B Values Std.	0.35*	1.08*	0.47
		[0.23; 0.46]	[0.47; 1.69]	[−0.15; 1.03]
	PMW Std.			1.53*
				[0.44; 2.57]
	Ideology Std.	0.22*	1.13*	0.78*
		[0.10; 0.35]	[0.53; 1.73]	[0.18; 1.42]
	Intercept[1]		0.13	0.19
			[−4.13; 3.73]	[−4.12; 3.65]
	Intercept[2]		2.33	2.41
			[−2.04; 5.84]	[−1.66; 6.09]
	Intercept[3]		3.50	3.59
			[−0.73; 7.17]	[−0.44; 7.33]
	Intercept[4]		4.76*	4.87*
			[0.44; 8.36]	[0.78; 8.57]
	Intercept[5]		6.54*	6.72*
			[2.26; 10.25]	[2.48; 10.33]
	Intercept[6]		8.47*	8.74*
			[4.22; 12.25]	[4.50; 12.41]
SD of Random Effects				
	Intercept_{EBEP}		4.25*	4.03*
			[1.48; 8.18]	[1.53; 7.93]
	$\text{Intercept}_{Subject}$		2.83*	2.44*
			[2.42; 3.26]	[2.06; 2.81]
	I Values Std_{EBEP}		0.69*	0.72*
			[0.01; 1.76]	[0.03; 1.83]
	B Values Std_{EBEP}		0.27*	0.28*
			[0.00; 0.84]	[0.00; 0.89]
	PMW Std_{EBEP}			0.80*
				[0.00; 2.10]
	Ideology Std_{EBEP}		0.28*	0.34*
			[0.00; 0.87]	[0.00; 1.03]

* 0 outside 95% highest posterior density interval. Estimates for Models 2 and 3 reported on log scale.

Ideology is coded such that higher values indicate stronger associations with Conservative ideology.

Supplementary Table 11

Study 3 Mediation Estimates for PMW for EBEP ≥ "Slightly justified"

EBEP	AME	ADE	Total
Facebook	**0.03** [0, 0.19]	**0.02** [0, 0.18]	**0.05** [0, 0.37]
Flyer	**0.03** [0, 0.18]	**0.02** [0, 0.18]	**0.05** [0, 0.34]
Yell	**0.03** [0, 0.19]	**0.03** [0, 0.17]	**0.06** [0, 0.36]
Assault	**0.03** [0, 0.18]	**0.03** [0, 0.15]	**0.05** [0, 0.31]
Assault	**0.03** [0, 0.18]	**0.03** [0, 0.17]	**0.05** [0, 0.34]

Supplementary Table 12

Study 3 Mediation Estimates for PMW for EBEP ≥ "Slightly justified" Adjusted for Ideology

EBEP	AME	ADE	Total
Facebook	**0.02** [0, 0.13]	0.02 [0, 0.14]	**0.03** [0, 0.26]
Flyer	**0.02** [0, 0.13]	0.02 [0, 0.12]	**0.03** [0, 0.24]
Yell	**0.02** [0, 0.11]	0.01 [0, 0.09]	**0.03** [0, 0.17]
Assault	**0.01** [0, 0.1]	0.01 [0, 0.09]	**0.03** [0, 0.18]
Marginal	**0.02** [0, 0.11]	0.01 [0, 0.1]	**0.03** [0, 0.19]

Supplementary Table 13

Study 3 Mediation Estimates for PMW at Each Response Level

EBEP	Response Level	AME	ADE	Total
Facebook	1	**-0.122** [-0.25, 0]	**-0.114** [-0.26, 0]	**-0.237** [-0.45, -0.01]
	2	0.046 [-0.15, 0.16]	0.044 [-0.13, 0.17]	0.089 [-0.27, 0.3]
	3	0.028 [-0.07, 0.1]	0.026 [-0.06, 0.09]	0.055 [-0.13, 0.17]
	4	0.022 [-0.04, 0.1]	0.02 [-0.04, 0.09]	0.042 [-0.1, 0.17]
	5	**0.017** [0, 0.11]	**0.015** [0, 0.1]	**0.032** [0, 0.21]
	6	**0.006** [0, 0.06]	**0.006** [0, 0.07]	**0.013** [0, 0.13]
	7	**0.003** [0, 0.03]	**0.003** [0, 0.04]	**0.007** [0, 0.08]
Flyer	1	**-0.123** [-0.25, 0]	**-0.12** [-0.25, 0]	**-0.243** [-0.45, -0.01]
	2	0.043 [-0.16, 0.17]	0.043 [-0.14, 0.17]	0.086 [-0.28, 0.31]
	3	0.029 [-0.07, 0.09]	0.029 [-0.06, 0.1]	0.058 [-0.13, 0.18]
	4	0.024 [-0.03, 0.11]	0.023 [-0.03, 0.1]	0.048 [-0.07, 0.19]
	5	**0.018** [0, 0.11]	**0.017** [0, 0.11]	**0.035** [0, 0.21]
	6	**0.006** [0, 0.05]	**0.005** [0, 0.05]	**0.011** [0, 0.11]
	7	**0.003** [0, 0.02]	**0.002** [0, 0.02]	**0.005** [0, 0.04]
Yell	1	**-0.121** [-0.25, 0]	**-0.113** [-0.25, 0]	**-0.233** [-0.44, 0]
	2	0.037 [-0.15, 0.17]	0.036 [-0.16, 0.16]	0.073 [-0.29, 0.3]
	3	0.029 [-0.07, 0.1]	0.027 [-0.06, 0.1]	0.056 [-0.13, 0.18]
	4	0.024 [-0.06, 0.1]	0.022 [-0.06, 0.1]	0.046 [-0.12, 0.2]
	5	0.018 [0, 0.12]	0.017 [-0.01, 0.12]	**0.035** [0, 0.22]
	6	**0.007** [0, 0.08]	**0.006** [0, 0.08]	**0.014** [0, 0.15]
	7	**0.004** [0, 0.05]	**0.004** [0, 0.04]	**0.009** [0, 0.08]
Assault	1	**-0.119** [-0.24, 0]	**-0.115** [-0.25, 0]	**-0.235** [-0.45, -0.01]
	2	0.037 [-0.16, 0.16]	0.036 [-0.17, 0.17]	0.073 [-0.3, 0.3]
	3	0.029 [-0.07, 0.1]	0.029 [-0.05, 0.1]	0.058 [-0.11, 0.19]
	4	0.025 [-0.05, 0.11]	0.025 [-0.04, 0.11]	0.05 [-0.08, 0.2]
	5	**0.018** [0, 0.12]	**0.017** [0, 0.1]	**0.035** [0, 0.2]
	6	**0.007** [0, 0.06]	**0.006** [0, 0.05]	**0.013** [0, 0.11]
	7	**0.003** [0, 0.02]	**0.003** [0, 0.02]	**0.006** [0, 0.06]

Supplementary Table 14

Study 3 Mediation Estimates for PMW at Each Response Level Adjusted for Ideology

EBEP	Response Level	AME	ADE	Total
Facebook	1	**-0.085** [-0.2, 0]	-0.077 [-0.22, 0.01]	**-0.162** [-0.36, -0.01]
	2	0.033 [-0.11, 0.13]	0.03 [-0.11, 0.14]	0.063 [-0.19, 0.25]
	3	0.021 [-0.05, 0.08]	0.018 [-0.05, 0.08]	0.039 [-0.1, 0.14]
	4	0.015 [-0.03, 0.07]	0.013 [-0.02, 0.07]	0.028 [-0.06, 0.13]
	5	0.011 [0, 0.08]	0.01 [0, 0.09]	**0.021** [0, 0.16]
	6	**0.004** [0, 0.05]	0.004 [0, 0.04]	**0.008** [0, 0.1]
	7	**0.002** [0, 0.01]	0.001 [0, 0.01]	**0.003** [0, 0.02]
Flyer	1	**-0.084** [-0.2, 0]	-0.076 [-0.21, 0.01]	**-0.161** [-0.36, -0.01]
	2	0.032 [-0.11, 0.13]	0.029 [-0.11, 0.14]	0.062 [-0.2, 0.24]
	3	0.02 [-0.05, 0.08]	0.018 [-0.05, 0.08]	0.038 [-0.1, 0.14]
	4	0.015 [-0.04, 0.07]	0.014 [-0.02, 0.07]	0.028 [-0.05, 0.14]
	5	**0.011** [0, 0.08]	0.01 [0, 0.08]	**0.021** [0, 0.14]
	6	**0.004** [0, 0.05]	0.004 [0, 0.04]	**0.008** [0, 0.08]
	7	**0.002** [0, 0.02]	0.001 [0, 0.01]	**0.003** [0, 0.03]
Yell	1	**-0.082** [-0.19, 0]	-0.075 [-0.21, 0.02]	**-0.157** [-0.34, 0]
	2	0.029 [-0.12, 0.13]	0.028 [-0.1, 0.14]	0.057 [-0.19, 0.23]
	3	0.021 [-0.04, 0.07]	0.02 [-0.03, 0.09]	0.042 [-0.07, 0.14]
	4	0.017 [-0.01, 0.08]	0.015 [-0.01, 0.07]	0.031 [-0.01, 0.13]
	5	0.01 [0, 0.08]	0.008 [-0.01, 0.06]	**0.019** [0, 0.12]
	6	**0.003** [0, 0.03]	0.003 [0, 0.02]	**0.006** [0, 0.04]
	7	**0.002** [0, 0.01]	0.001 [0, 0]	**0.003** [0, 0.01]
Assault	1	**-0.084** [-0.19, 0]	-0.074 [-0.2, 0]	**-0.159** [-0.34, -0.01]
	2	0.03 [-0.11, 0.13]	0.026 [-0.12, 0.13]	0.056 [-0.2, 0.23]
	3	0.023 [-0.04, 0.08]	0.02 [-0.04, 0.08]	0.043 [-0.07, 0.14]
	4	0.017 [-0.02, 0.08]	0.016 [-0.01, 0.08]	0.032 [-0.01, 0.14]
	5	0.01 [0, 0.07]	0.009 [0, 0.07]	**0.019** [0, 0.12]
	6	**0.003** [0, 0.03]	0.002 [0, 0.02]	**0.006** [0, 0.04]
	7	**0.001** [0, 0.01]	0.001 [0, 0.01]	**0.002** [0, 0.01]

Supplementary Table 15

Study 4 Model Estimates.

Fixed Effects		Model 1	Model 2	Model 3
		PMW Std.	EBEP J	EBEP J
	Intercept	-0.24^{*}		
		[−0.40; −0.09]		
	High Moral Threat	0.50^{*}	1.44^{*}	0.28
		[0.27; 0.72]	[0.15; 2.78]	[−0.87; 1.55]
	PMW Std.			2.21^{*}
				[1.48; 2.94]
	Intercept[1]		0.71	0.17
			[−3.55; 4.43]	[−3.63; 3.76]
	Intercept[2]		2.65	2.10
			[−1.46; 6.56]	[−1.64; 5.74]
	Intercept[3]		3.65	3.10
			[−0.66; 7.34]	[−0.76; 6.67]
	Intercept[4]		5.19^{*}	4.68^{*}
			[0.89; 8.93]	[0.74; 8.19]
	Intercept[5]		6.70^{*}	6.25^{*}
			[2.28; 10.34]	[2.23; 9.69]
	Intercept[6]		9.14^{*}	8.83^{*}
			[4.74; 12.87]	[4.98; 12.48]
SD of Random Effects				
	$\text{Intercept}_{\text{Subject}}$		3.79^{*}	2.99^{*}
			[3.27; 4.37]	[2.55; 3.45]
	$\text{Intercept}_{\text{EBEP}}$		4.04^{*}	3.78^{*}
			[1.36; 8.12]	[1.28; 7.48]
	$\text{High Moral Threat}_{\text{EBEP}}$		0.62^{*}	0.71^{*}
			[0.00; 1.76]	[0.00; 2.06]
	$\text{PMW Std}_{\text{EBEP}}$			0.47^{*}
				[0.00; 1.29]

* 0 outside 95% highest posterior density interval. Estimates for Models 2 and 3 reported on log scale.

Supplementary Table 16

Study 4 Model Estimates Adjusted for Ideology

Fixed Effects		Model 1	Model 2	Model 3
		PMW Std.	EBEP J	EBEP J
	Intercept	−0.23*		
		[−0.38; −0.08]		
	High Moral Threat	0.47*	1.48*	0.51
		[0.25; 0.68]	[0.27; 2.68]	[−0.72; 1.70]
	Ideology Std.	0.32*	1.88*	1.22*
		[0.21; 0.43]	[1.15; 2.64]	[0.38; 1.96]
	PMW Std.			1.83*
				[1.03; 2.63]
	Intercept[1]		0.67	0.24
			[−3.68; 4.32]	[−3.55; 3.67]
	Intercept[2]		2.65	2.21
			[−1.68; 6.31]	[−1.55; 5.65]
	Intercept[3]		3.65	3.22
			[−0.65; 7.34]	[−0.56; 6.65]
	Intercept[4]		5.26*	4.86*
			[1.00; 9.03]	[1.15; 8.39]
	Intercept[5]		6.80*	6.46*
			[2.51; 10.57]	[2.62; 9.94]
	Intercept[6]		9.23*	9.00*
			[4.83; 12.97]	[5.13; 12.56]
SD of Random Effects				
	$\text{Intercept}_{Subject}$		3.40*	2.85*
			[2.89; 3.93]	[2.41; 3.29]
	Intercept_{EBEP}		4.12*	3.85*
			[1.33; 8.06]	[1.21; 7.52]
	$\text{High Moral Threat}_{EBEP}$		0.60*	0.66*
			[0.00; 1.73]	[0.00; 1.97]
	$\text{Ideology Std}_{EBEP}$		0.46*	0.51*
			[0.00; 1.26]	[0.00; 1.41]
	PMW Std_{EBEP}			0.50*
				[0.00; 1.40]

* 0 outside 95% highest posterior density interval. Estimates for Models 2 and 3 reported on log scale. Ideology is coded such that higher values indicate stronger associations with Conservative ideology.

Supplementary Table 17

Study 4 Mediation Estimates for PMW for EBEP ≥ "Slightly justified"

EBEP	AME	ADE	Total
Facebook	**0.03** [0, 0.24]	0.01 [-0.03, 0.08]	**0.04** [0, 0.28]
Flyer	**0.03** [0, 0.2]	0.01 [-0.02, 0.11]	**0.04** [0, 0.26]
Yell	**0.03** [0, 0.19]	0.01 [-0.02, 0.07]	**0.03** [0, 0.23]
Assault	**0.03** [0, 0.21]	0.01 [-0.02, 0.11]	**0.04** [0, 0.25]
Marginal	**0.03** [0, 0.2]	0.01 [-0.02, 0.08]	**0.04** [0, 0.24]

NOTE: Cell values represent posterior means and 95% CIs. Bold entries indicate that the CI does not overlap with zero. Because EBEP is ordinal, mediation is estimated at each level of the variable. Here, however, results are summarized to reflect the indirect, direct, and total effects on the probability of an EBEP being rated as at least 'Slightly Justified' marginalized across EBEP items.

Supplementary Table 18

Study 4 Mediation Estimates for PMW for EBEP ≥ "Slightly justified" Adjusted for Ideology

EBEP	AME	ADE	Total
Facebook	**0.02** [0, 0.19]	0.02 [-0.01, 0.15]	**0.04** [0, 0.32]
Flyer	**0.03** [0, 0.2]	0.01 [-0.02, 0.13]	**0.04** [0, 0.32]
Yell	**0.02** [0, 0.17]	0.01 [-0.01, 0.11]	**0.03** [0, 0.23]
Assault	**0.02** [0, 0.15]	0.01 [-0.01, 0.12]	**0.03** [0, 0.23]
Marginal	**0.02** [0, 0.18]	0.01 [-0.01, 0.11]	**0.03** [0, 0.26]

NOTE: Cell values represent posterior means and 95% CIs. Bold entries indicate that the CI does not overlap with zero. Because EBEP is ordinal, mediation is estimated at each level of the variable. Here, however, results are summarized to reflect the indirect, direct, and total effects on the probability of an EBEP being rated as at least 'Slightly Justified' marginalized across EBEP items. All effects were estimated with standardized political ideology set to its mean.

Supplementary Table 19

Study 4 Mediation Estimates for PMW at Each Response Level

EBEP	Response Level	AME	ADE	Total
Facebook	1	**-0.185** [-0.37, -0.01]	-0.045 [-0.29, 0.15]	-0.23 [-0.56, 0]
	2	0.072 [-0.19, 0.23]	0.021 [-0.1, 0.18]	0.092 [-0.24, 0.34]
	3	0.041 [-0.08, 0.13]	0.009 [-0.06, 0.09]	0.049 [-0.1, 0.18]
	4	0.041 [-0.05, 0.17]	0.008 [-0.05, 0.09]	0.049 [-0.07, 0.22]
	5	**0.019** [0, 0.14]	0.003 [-0.02, 0.04]	**0.023** [0, 0.17]
	6	**0.01** [0, 0.11]	0.003 [-0.01, 0.02]	**0.014** [0, 0.13]
	7	**0.002** [0, 0.01]	0.001 [0, 0]	**0.003** [0, 0.02]
Flyer	1	**-0.185** [-0.37, -0.01]	-0.047 [-0.29, 0.12]	**-0.232** [-0.55, 0]
	2	0.07 [-0.19, 0.23]	0.018 [-0.13, 0.16]	0.088 [-0.25, 0.33]
	3	0.042 [-0.07, 0.12]	0.01 [-0.05, 0.09]	0.052 [-0.09, 0.18]
	4	0.044 [-0.01, 0.17]	0.01 [-0.05, 0.12]	0.054 [-0.01, 0.24]
	5	**0.018** [0, 0.13]	0.004 [-0.02, 0.06]	**0.022** [0, 0.16]
	6	**0.008** [0, 0.07]	0.004 [0, 0.04]	**0.011** [0, 0.1]
	7	**0.003** [0, 0.01]	0.001 [0, 0]	**0.004** [0, 0.02]
Yell	1	**-0.18** [-0.36, -0.01]	-0.046 [-0.29, 0.15]	**-0.225** [-0.54, 0]
	2	0.067 [-0.19, 0.22]	0.016 [-0.12, 0.15]	0.082 [-0.25, 0.31]
	3	0.042 [-0.07, 0.13]	0.011 [-0.04, 0.1]	0.053 [-0.08, 0.18]
	4	**0.044** [0, 0.17]	0.012 [-0.04, 0.11]	0.056 [-0.01, 0.24]
	5	**0.018** [0, 0.12]	0.005 [-0.01, 0.05]	**0.023** [0, 0.15]
	6	**0.007** [0, 0.06]	0.002 [0, 0.02]	**0.01** [0, 0.07]
	7	**0.002** [0, 0.01]	0 [0, 0]	**0.002** [0, 0.01]
Assault	1	**-0.185** [-0.37, -0.01]	-0.049 [-0.31, 0.16]	**-0.234** [-0.58, 0]
	2	0.063 [-0.18, 0.23]	0.013 [-0.17, 0.16]	0.076 [-0.25, 0.34]
	3	0.046 [-0.07, 0.13]	0.013 [-0.06, 0.09]	0.059 [-0.08, 0.19]
	4	0.046 [-0.04, 0.17]	0.013 [-0.04, 0.11]	0.06 [-0.03, 0.24]
	5	**0.019** [0, 0.11]	0.006 [-0.01, 0.08]	**0.025** [0, 0.15]
	6	**0.009** [0, 0.09]	0.003 [0, 0.03]	**0.012** [0, 0.11]
	7	**0.002** [0, 0.01]	0 [0, 0]	**0.002** [0, 0.02]

Supplementary Table 20

Study 4 Mediation Estimates for PMW at Each Response Level Adjusted for Ideology

EBEP	Response Level	AME	ADE	Total
Facebook	1	**-0.137** [-0.3, 0]	-0.084 [-0.38, 0.1]	-0.221 [-0.57, 0]
	2	0.057 [-0.15, 0.19]	0.033 [-0.17, 0.22]	0.09 [-0.26, 0.34]
	3	0.028 [-0.07, 0.1]	0.017 [-0.05, 0.11]	0.045 [-0.11, 0.18]
	4	0.029 [-0.06, 0.13]	0.017 [-0.06, 0.14]	0.046 [-0.1, 0.25]
	5	**0.014** [0, 0.11]	0.009 [-0.02, 0.09]	**0.022** [0, 0.19]
	6	**0.008** [0, 0.09]	0.007 [0, 0.06]	**0.015** [0, 0.17]
	7	**0.001** [0, 0.01]	0.001 [0, 0.01]	**0.002** [0, 0.03]
Flyer	1	**-0.139** [-0.3, 0]	-0.073 [-0.36, 0.16]	-0.212 [-0.54, 0]
	2	0.051 [-0.15, 0.19]	0.028 [-0.13, 0.19]	0.08 [-0.24, 0.33]
	3	0.031 [-0.07, 0.11]	0.016 [-0.06, 0.11]	0.047 [-0.11, 0.19]
	4	0.031 [-0.08, 0.14]	0.016 [-0.07, 0.12]	0.047 [-0.11, 0.22]
	5	**0.014** [0, 0.11]	0.007 [-0.02, 0.08]	**0.021** [0, 0.17]
	6	**0.009** [0, 0.11]	0.004 [0, 0.05]	**0.013** [0, 0.15]
	7	**0.002** [0, 0.02]	0.002 [0, 0.01]	**0.004** [0, 0.02]
Yell	1	**-0.145** [-0.3, -0.01]	-0.079 [-0.32, 0.1]	**-0.224** [-0.52, 0]
	2	0.057 [-0.15, 0.19]	0.029 [-0.14, 0.19]	0.086 [-0.26, 0.34]
	3	0.033 [-0.06, 0.1]	0.018 [-0.04, 0.11]	0.051 [-0.08, 0.18]
	4	0.033 [-0.03, 0.14]	0.018 [-0.03, 0.13]	0.051 [-0.05, 0.23]
	5	**0.014** [0, 0.1]	0.008 [-0.01, 0.07]	**0.022** [0, 0.14]
	6	**0.007** [0, 0.06]	0.004 [0, 0.03]	**0.011** [0, 0.09]
	7	**0.001** [0, 0.01]	0.001 [0, 0]	**0.002** [0, 0.01]
Assault	1	**-0.14** [-0.3, -0.01]	-0.077 [-0.33, 0.09]	**-0.216** [-0.54, 0]
	2	0.054 [-0.15, 0.18]	0.032 [-0.11, 0.18]	0.086 [-0.22, 0.32]
	3	0.034 [-0.05, 0.1]	0.017 [-0.05, 0.11]	0.051 [-0.08, 0.18]
	4	0.033 [-0.01, 0.14]	0.017 [-0.03, 0.13]	0.05 [-0.01, 0.22]
	5	**0.013** [0, 0.09]	0.007 [-0.01, 0.08]	**0.02** [0, 0.15]
	6	**0.005** [0, 0.05]	0.003 [0, 0.04]	**0.008** [0, 0.08]
	7	**0.001** [0, 0.01]	0.001 [0, 0]	**0.002** [0, 0.01]

NOTE: Cell values represent posterior means and 95% CIs. Bold entries indicate that the CI does not overlap with zero. All effects were estimated with standardized political ideology set to its mean.

Supplementary Table 21

Study 5 Model Estimates for Binding Values Violation Condition

Fixed Effects		Model 1 PMW Std.	Model 2 EBEP
	Intercept	-0.93^*	
		$[-1.00; -0.85]$	
	Binding Values Threat	1.38^*	1.04
		$[1.28; 1.49]$	$[-0.79; 2.90]$
	Binding Values Std.	0.12^*	1.38^*
		$[0.02; 0.22]$	$[0.37; 2.39]$
	Individualizing Values Std.	-0.10^*	-1.30^*
		$[-0.19; -0.01]$	$[-2.26; -0.33]$
	Ideology Std.	-0.04	-0.50
		$[-0.10; 0.01]$	$[-1.24; 0.25]$
	Religiosity Std.	0.04	0.33
		$[-0.02; 0.10]$	$[-0.07; 0.77]$
	Binding Values Threat x	0.30^*	-0.44
	Binding Values Std.	$[0.16; 0.43]$	$[-1.62; 0.71]$
	Binding Values Threat x	-0.07	0.39
	Individualizing Values Std.	$[-0.21; 0.06]$	$[-0.85; 1.60]$
	Intercept[1]		-0.42
			$[-2.34; 1.44]$
	Intercept[2]		0.95
			$[-0.96; 2.81]$
	Intercept[3]		1.69
			$[-0.29; 3.49]$
	Intercept[4]		3.39^*
			$[1.39; 5.18]$
	Intercept[5]		4.55^*
			$[2.54; 6.36]$
	Intercept[6]		6.32^*
			$[4.41; 8.21]$
	PMW Std.		2.43^*
			$[1.84; 2.95]$
	Binding Values Std. x PMW Std.		0.62^*
			$[0.06; 1.23]$
	Individualizing Values Std. x PMW Std.		-0.22
			$[-0.82; 0.34]$
SD of Random Effects			
	$\text{Intercept}_{Subject}$		3.14* [2.84; 3.46]
	Intercept_{EBEP}		1.65* [0.59; 4.45]
	B Threat_{EBEP}		1.50* [0.49; 4.14]
	PMW Std._{EBEP}		0.28* [0.01; 1.33]
	$\text{I Values Std}_{EBEP}$		0.51* [0.03; 1.99]
	$\text{B Values Std}_{EBEP}$		0.63* [0.02; 1.98]
	$\text{Ideology Std}_{EBEP}$		0.57* [0.14; 1.90]
	Relig Std_{EBEP}		0.20* [0.01; 0.91]
	$\text{B Threat x I Values Std}_{EBEP}$		0.54* [0.02; 2.18]
	$\text{B Threat x B Values Std}_{EBEP}$		0.46* [0.01; 1.86]
	$\text{I Threat x PMW Std.}_{EBEP}$		0.20 [0.01; 0.88]

* 0 outside 95% highest posterior density interval

Supplementary Table 22

Study 5 Moderated Mediation Estimates for Binding Values Threat and PMW at Low Binding Values

EBEP	Response Level	AME	ADE	Total
Facebook	1	**-0.348** [-0.57, -0.12]	**-0.346** [-0.65, -0.04]	**-0.693** [-0.96, -0.41]
	2	**0.183** [0.07, 0.27]	**0.175** [0.01, 0.28]	**0.358** [0.15, 0.48]
	3	**0.071** [0.02, 0.14]	**0.071** [0.01, 0.15]	**0.142** [0.07, 0.23]
	4	**0.074** [0.02, 0.19]	**0.076** [0.01, 0.23]	**0.15** [0.06, 0.31]
	5	**0.014** [0, 0.04]	**0.015** [0, 0.05]	**0.029** [0.01, 0.08]
	6	**0.005** [0, 0.02]	**0.006** [0, 0.02]	**0.012** [0, 0.03]
	7	**0.001** [0, 0]	**0.001** [0, 0]	**0.003** [0, 0.01]
Flyer	1	**-0.288** [-0.52, -0.09]	**-0.276** [-0.56, -0.04]	**-0.563** [-0.86, -0.26]
	2	**0.17** [0.06, 0.26]	**0.159** [0.02, 0.27]	**0.329** [0.15, 0.45]
	3	**0.054** [0.01, 0.12]	**0.052** [0.01, 0.13]	**0.106** [0.04, 0.2]
	4	**0.051** [0.01, 0.13]	**0.051** [0, 0.17]	**0.102** [0.03, 0.24]
	5	**0.009** [0, 0.03]	**0.009** [0, 0.04]	**0.018** [0.01, 0.05]
	6	**0.003** [0, 0.01]	**0.004** [0, 0.01]	**0.007** [0, 0.02]
	7	**0.001** [0, 0]	**0.001** [0, 0]	**0.002** [0, 0]
Yell	1	**-0.211** [-0.44, -0.06]	**-0.162** [-0.45, -0.01]	**-0.373** [-0.66, -0.16]
	2	**0.136** [0.04, 0.25]	**0.105** [0, 0.25]	**0.241** [0.12, 0.38]
	3	**0.036** [0.01, 0.09]	**0.027** [0, 0.09]	**0.063** [0.02, 0.13]
	4	**0.031** [0.01, 0.09]	**0.024** [0, 0.08]	**0.055** [0.02, 0.13]
	5	**0.005** [0, 0.02]	**0.004** [0, 0.01]	**0.009** [0, 0.02]
	6	**0.002** [0, 0.01]	**0.002** [0, 0.01]	**0.004** [0, 0.01]
	7	**0** [0, 0]	**0** [0, 0]	**0.001** [0, 0]
Assault	1	**-0.088** [-0.24, -0.02]	-0.007 [-0.05, 0.02]	**-0.095** [-0.25, -0.03]
	2	**0.062** [0.01, 0.16]	0.005 [-0.02, 0.04]	**0.067** [0.02, 0.17]
	3	**0.013** [0, 0.04]	0.001 [0, 0.01]	**0.014** [0, 0.04]
	4	**0.01** [0, 0.03]	0.001 [0, 0.01]	**0.011** [0, 0.03]
	5	**0.002** [0, 0.01]	0 [0, 0]	**0.002** [0, 0.01]
	6	**0.001** [0, 0]	0 [0, 0]	**0.001** [0, 0]
	7	**0** [0, 0]	0 [0, 0]	**0** [0, 0]

Supplementary Table 23

Study 5 Moderated Mediation Estimates for Binding Values Threat and PMW at High Binding Values

EBEP	Response Level	AME	ADE	Total
Facebook	1	**-0.482** [-0.75, -0.21]	-0.231 [-0.5, 0.05]	**-0.713** [-1.18, -0.23]
	2	**-0.263** [-0.34, -0.11]	0 [-0.16, 0.14]	-0.263 [-0.46, 0.02]
	3	-0.068 [-0.16, 0.03]	0.054 [-0.03, 0.12]	-0.014 [-0.18, 0.14]
	4	0.077 [-0.16, 0.32]	0.121 [-0.02, 0.28]	0.198 [-0.14, 0.56]
	5	**0.208** [0.06, 0.29]	0.037 [0, 0.12]	**0.245** [0.07, 0.37]
	6	**0.333** [0.15, 0.44]	0.017 [0, 0.06]	**0.35** [0.17, 0.45]
	7	**0.195** [0.04, 0.53]	0.004 [0, 0.01]	**0.198** [0.04, 0.53]
Flyer	1	**-0.57** [-0.81, -0.28]	-0.219 [-0.53, 0.14]	**-0.788** [-1.24, -0.27]
	2	**-0.22** [-0.33, -0.06]	0.041 [-0.13, 0.17]	-0.179 [-0.39, 0.1]
	3	-0.036 [-0.14, 0.07]	0.054 [-0.04, 0.13]	0.017 [-0.16, 0.18]
	4	0.145 [-0.1, 0.36]	0.089 [-0.03, 0.27]	0.233 [-0.11, 0.57]
	5	**0.228** [0.09, 0.3]	0.023 [-0.01, 0.1]	**0.251** [0.09, 0.35]
	6	**0.3** [0.11, 0.43]	0.01 [0, 0.05]	**0.311** [0.13, 0.44]
	7	**0.152** [0.03, 0.44]	0.002 [0, 0.01]	**0.155** [0.03, 0.44]
Yell	1	**-0.693** [-0.86, -0.42]	-0.173 [-0.46, 0.13]	**-0.866** [-1.2, -0.38]
	2	-0.134 [-0.29, 0.04]	0.073 [-0.09, 0.2]	-0.061 [-0.34, 0.22]
	3	0.016 [-0.08, 0.12]	0.038 [-0.03, 0.11]	0.054 [-0.1, 0.21]
	4	**0.246** [0.02, 0.4]	0.046 [-0.03, 0.17]	**0.292** [0.01, 0.5]
	5	**0.242** [0.12, 0.3]	0.01 [0, 0.05]	**0.252** [0.14, 0.32]
	6	**0.235** [0.07, 0.41]	0.005 [0, 0.02]	**0.24** [0.08, 0.41]
	7	**0.089** [0.02, 0.28]	0.001 [0, 0]	**0.09** [0.02, 0.29]
Assault	1	**-0.823** [-0.92, -0.68]	0.042 [-0.08, 0.17]	**-0.781** [-0.92, -0.56]
	2	0.045 [-0.12, 0.23]	-0.029 [-0.11, 0.06]	0.015 [-0.22, 0.27]
	3	**0.106** [0.01, 0.18]	-0.006 [-0.03, 0.01]	0.099 [-0.01, 0.19]
	4	**0.347** [0.19, 0.42]	-0.005 [-0.02, 0.01]	**0.342** [0.18, 0.42]
	5	**0.181** [0.06, 0.29]	-0.001 [0, 0]	**0.18** [0.06, 0.29]
	6	**0.114** [0.02, 0.3]	0 [0, 0]	**0.114** [0.02, 0.3]
	7	**0.031** [0, 0.1]	0 [0, 0]	**0.031** [0, 0.1]

Supplementary Table 24

Study 5 Model Estimates for Individualizing Values Violation Condition

Fixed Effects		Model 1 PMW Std.	Model 2 EBEP
	Intercept	−0.93*	
		[−1.01; −0.85]	
	Individualizing Values Threat	1.39*	1.43
		[1.28; 1.50]	[−0.70; 3.41]
	Binding Values Std.	0.14*	0.93
		[0.04; 0.24]	[−0.11; 1.90]
	Individualizing Values Std.	−0.11*	−0.93*
		[−0.22; −0.01]	[−1.80; −0.03]
	Ideology Std.	−0.07*	−0.46*
		[−0.13; −0.01]	[−0.80; −0.13]
	Religiosity Std.	0.01	0.16
		[−0.06; 0.07]	[−0.18; 0.54]
	Individualizing Values Threat x	−0.09	−0.12
	Binding Values Std.	[−0.22; 0.04]	[−1.14; 0.89]
	Individualizing Values Threat x	0.30*	0.41
	Individualizing Values Std.	[0.17; 0.43]	[−0.97; 1.62]
	Intercept[1]		−0.55
			[−2.26; 1.13]
	Intercept[2]		0.55
			[−1.13; 2.26]
	Intercept[3]		1.43
			[−0.24; 3.18]
	Intercept[4]		3.01*
			[1.24; 4.66]
	Intercept[5]		4.13*
			[2.44; 5.87]
	Intercept[6]		5.62*
			[3.87; 7.30]
	PMW Std.		2.38*
			[1.88; 2.92]
	Binding Values Std. x PMW Std.		0.16
			[−0.33; 0.61]
	Individualizing Values Std. x PMW Std.		0.10
			[−0.35; 0.54]
SD of Random Effects			
	$\text{Intercept}_{Subject}$		2.71* [2.44; 2.99]
	Intercept_{EBEP}		1.50* [0.54; 4.20]
	I Threat_{EBEP}		1.79* [0.66; 4.68]
	PMW Std._{EBEP}		0.28* [0.01; 1.33]
	$\text{I Values Std}_{EBEP}$		0.48* [0.02; 1.91]
	$\text{B Values Std}_{EBEP}$		0.63* [0.07; 2.37]
	$\text{Ideology Std}_{EBEP}$		0.15 [0.00; 0.62]
	Relig Std_{EBEP}		0.16 [0.00; 0.77]
	$\text{I Threat x I Values Std}_{EBEP}$		0.85* [0.15; 2.77]
	$\text{I Threat x B Values Std}_{EBEP}$		0.38* [0.01; 1.61]
	$\text{I Threat x PMW Std.}_{EBEP}$		0.16 [0.00; 0.71]

* 0 outside 95% highest posterior density interval

Supplementary Table 25

Study 5 Moderated Mediation Estimates for Individualizing Values Threat and PMW at Low Binding Values

EBEP	Response Level	AME	ADE	Total
Facebook	1	**-0.626** [-0.79, -0.37]	**-0.403** [-0.66, -0.1]	**-1.028** [-1.26, -0.69]
	2	0.143 [-0.04, 0.24]	**0.153** [0.03, 0.23]	**0.296** [0.02, 0.46]
	3	**0.166** [0.07, 0.21]	**0.11** [0.02, 0.19]	**0.276** [0.14, 0.37]
	4	**0.216** [0.07, 0.35]	**0.102** [0.01, 0.25]	**0.318** [0.13, 0.51]
	5	**0.064** [0.01, 0.16]	**0.024** [0, 0.07]	**0.088** [0.03, 0.19]
	6	**0.028** [0.01, 0.08]	**0.01** [0, 0.03]	**0.038** [0.01, 0.1]
	7	**0.009** [0, 0.03]	**0.003** [0, 0.01]	**0.012** [0, 0.03]
Flyer	1	**-0.585** [-0.77, -0.23]	**-0.395** [-0.68, -0.11]	**-0.98** [-1.24, -0.56]
	2	**0.179** [0, 0.26]	**0.166** [0.02, 0.24]	**0.345** [0.01, 0.49]
	3	**0.158** [0.04, 0.22]	**0.105** [0.02, 0.19]	**0.262** [0.08, 0.37]
	4	**0.174** [0.04, 0.33]	**0.09** [0.01, 0.24]	**0.265** [0.09, 0.45]
	5	**0.047** [0.01, 0.13]	**0.021** [0, 0.07]	**0.068** [0.02, 0.16]
	6	**0.021** [0, 0.07]	**0.009** [0, 0.03]	**0.03** [0.01, 0.08]
	7	**0.007** [0, 0.02]	**0.003** [0, 0.01]	**0.01** [0, 0.03]
Yell	1	**-0.505** [-0.75, -0.2]	**-0.141** [-0.39, 0]	**-0.646** [-0.91, -0.33]
	2	**0.203** [0.06, 0.26]	0.077 [-0.01, 0.18]	**0.28** [0.09, 0.39]
	3	**0.135** [0.04, 0.21]	0.032 [0, 0.1]	**0.167** [0.08, 0.26]
	4	**0.121** [0.03, 0.28]	**0.023** [0, 0.09]	**0.144** [0.05, 0.3]
	5	**0.029** [0.01, 0.08]	**0.005** [0, 0.02]	**0.034** [0.01, 0.09]
	6	**0.013** [0, 0.04]	**0.002** [0, 0.01]	**0.015** [0, 0.04]
	7	**0.004** [0, 0.01]	**0.001** [0, 0]	**0.005** [0, 0.01]
Assault	1	**-0.312** [-0.6, -0.06]	-0.004 [-0.05, 0.03]	**-0.317** [-0.59, -0.08]
	2	**0.166** [0.04, 0.26]	0.002 [-0.02, 0.03]	**0.168** [0.04, 0.26]
	3	**0.077** [0.01, 0.17]	0.001 [-0.01, 0.01]	**0.078** [0.02, 0.16]
	4	**0.054** [0.01, 0.15]	0.001 [0, 0.01]	**0.054** [0.01, 0.15]
	5	**0.011** [0, 0.03]	0 [0, 0]	**0.011** [0, 0.03]
	6	**0.004** [0, 0.01]	0 [0, 0]	**0.004** [0, 0.01]
	7	**0.001** [0, 0]	0 [0, 0]	**0.001** [0, 0]

Supplementary Table 26

Study 5 Moderated Mediation Estimates for Individualizing Values Threat and PMW at High Binding Values

EBEP	Response Level	AME	ADE	Total
Facebook	1	**-0.416** [-0.65, -0.17]	**-0.33** [-0.55, -0.12]	**-0.746** [-1.17, -0.3]
	2	**-0.186** [-0.26, -0.04]	-0.091 [-0.21, 0.08]	**-0.276** [-0.43, 0]
	3	-0.044 [-0.16, 0.11]	0.038 [-0.11, 0.14]	-0.005 [-0.24, 0.23]
	4	0.194 [-0.04, 0.33]	**0.201** [0.05, 0.3]	**0.395** [0.05, 0.62]
	5	**0.209** [0.11, 0.27]	**0.104** [0.02, 0.22]	**0.313** [0.19, 0.43]
	6	**0.166** [0.06, 0.29]	**0.057** [0.01, 0.17]	**0.222** [0.1, 0.37]
	7	**0.077** [0.02, 0.19]	**0.021** [0, 0.08]	**0.098** [0.03, 0.22]
Flyer	1	**-0.499** [-0.69, -0.21]	**-0.412** [-0.63, -0.15]	**-0.911** [-1.26, -0.41]
	2	-0.134 [-0.24, 0.06]	-0.049 [-0.19, 0.12]	-0.183 [-0.4, 0.14]
	3	0.02 [-0.13, 0.16]	0.079 [-0.08, 0.16]	0.099 [-0.16, 0.3]
	4	**0.257** [0.03, 0.35]	**0.22** [0.05, 0.32]	**0.477** [0.14, 0.64]
	5	**0.185** [0.06, 0.27]	**0.094** [0.02, 0.21]	**0.279** [0.13, 0.4]
	6	**0.122** [0.03, 0.26]	**0.049** [0.01, 0.16]	**0.17** [0.06, 0.32]
	7	**0.049** [0.01, 0.13]	**0.018** [0, 0.06]	**0.068** [0.02, 0.17]
Yell	1	**-0.574** [-0.73, -0.31]	-0.265 [-0.51, 0.02]	**-0.838** [-1.14, -0.4]
	2	-0.064 [-0.21, 0.13]	0.057 [-0.07, 0.15]	-0.008 [-0.22, 0.27]
	3	0.081 [-0.05, 0.18]	0.078 [-0.02, 0.16]	0.159 [-0.04, 0.32]
	4	**0.287** [0.14, 0.36]	0.092 [-0.01, 0.23]	**0.379** [0.17, 0.52]
	5	**0.151** [0.05, 0.25]	0.024 [0, 0.09]	**0.175** [0.07, 0.27]
	6	**0.086** [0.02, 0.2]	0.01 [0, 0.04]	**0.097** [0.03, 0.21]
	7	**0.033** [0.01, 0.09]	0.003 [0, 0.01]	**0.036** [0.01, 0.09]
Assault	1	**-0.628** [-0.78, -0.3]	0.004 [-0.15, 0.15]	**-0.624** [-0.8, -0.27]
	2	0.117 [-0.05, 0.23]	-0.004 [-0.09, 0.08]	0.113 [-0.11, 0.28]
	3	**0.163** [0.05, 0.21]	-0.001 [-0.03, 0.04]	**0.162** [0.04, 0.22]
	4	**0.23** [0.06, 0.35]	0 [-0.02, 0.03]	**0.231** [0.07, 0.35]
	5	**0.072** [0.01, 0.16]	0 [0, 0.01]	**0.072** [0.01, 0.16]
	6	**0.033** [0, 0.09]	0 [0, 0]	**0.033** [0.01, 0.09]
	7	**0.013** [0, 0.03]	0 [0, 0]	**0.013** [0, 0.03]

Supplementary Table 27

Study 5 Moderated Mediation Estimates for Individualizing Values Threat and PMW at Low Individualizing Values

EBEP	Response Level	AME	ADE	Total
Facebook	1	**-0.36** [-0.56, -0.16]	**-0.281** [-0.49, -0.01]	**-0.641** [-1.01, -0.22]
	2	**-0.15** [-0.23, 0]	-0.084 [-0.2, 0.07]	-0.234 [-0.38, 0.05]
	3	-0.004 [-0.14, 0.13]	0.034 [-0.09, 0.13]	0.029 [-0.19, 0.25]
	4	**0.226** [0.05, 0.31]	0.179 [-0.02, 0.29]	**0.405** [0.1, 0.58]
	5	**0.158** [0.07, 0.23]	**0.088** [0, 0.21]	**0.245** [0.13, 0.36]
	6	**0.096** [0.03, 0.2]	**0.047** [0, 0.15]	**0.143** [0.05, 0.28]
	7	**0.035** [0.01, 0.09]	**0.017** [0, 0.06]	**0.053** [0.02, 0.12]
Flyer	1	**-0.428** [-0.59, -0.23]	**-0.361** [-0.59, -0.1]	**-0.788** [-1.13, -0.38]
	2	-0.099 [-0.21, 0.06]	-0.051 [-0.19, 0.08]	-0.151 [-0.35, 0.1]
	3	0.056 [-0.08, 0.15]	0.072 [-0.06, 0.15]	0.127 [-0.09, 0.29]
	4	**0.253** [0.14, 0.32]	**0.2** [0.04, 0.31]	**0.452** [0.25, 0.59]
	5	**0.127** [0.05, 0.21]	**0.083** [0.01, 0.2]	**0.21** [0.1, 0.34]
	6	**0.068** [0.02, 0.16]	**0.042** [0, 0.13]	**0.111** [0.05, 0.23]
	7	**0.024** [0.01, 0.06]	**0.015** [0, 0.05]	**0.039** [0.01, 0.1]
Yell	1	**-0.523** [-0.75, -0.18]	**-0.135** [-0.38, -0.01]	**-0.658** [-0.92, -0.29]
	2	**0.202** [0.03, 0.26]	0.073 [0, 0.18]	**0.275** [0.06, 0.39]
	3	**0.141** [0.03, 0.21]	**0.031** [0, 0.1]	**0.171** [0.06, 0.25]
	4	**0.131** [0.03, 0.29]	**0.022** [0, 0.09]	**0.153** [0.04, 0.33]
	5	**0.031** [0, 0.09]	**0.005** [0, 0.02]	**0.037** [0.01, 0.1]
	6	**0.013** [0, 0.04]	**0.003** [0, 0.01]	**0.016** [0, 0.05]
	7	**0.004** [0, 0.01]	**0.001** [0, 0]	**0.005** [0, 0.01]
Assault	1	**-0.314** [-0.61, -0.08]	-0.006 [-0.05, 0.02]	**-0.32** [-0.61, -0.09]
	2	**0.167** [0.05, 0.26]	0.003 [-0.01, 0.03]	**0.17** [0.06, 0.26]
	3	**0.077** [0.02, 0.17]	0.001 [0, 0.01]	**0.078** [0.02, 0.17]
	4	**0.055** [0.01, 0.15]	0.001 [0, 0.01]	**0.055** [0.01, 0.15]
	5	**0.011** [0, 0.03]	0 [0, 0]	**0.011** [0, 0.03]
	6	**0.004** [0, 0.01]	0 [0, 0]	**0.004** [0, 0.01]
	7	**0.001** [0, 0]	0 [0, 0]	**0.001** [0, 0]

Supplementary Table 28

Study 5 Moderated Mediation Estimates for Individualizing Values Threat and PMW at High Individualizing Values

EBEP	Response Level	AME	ADE	Total
Facebook	1	**-0.745** [-0.87, -0.5]	**-0.447** [-0.74, -0.11]	**-1.192** [-1.45, -0.85]
	2	0.108 [-0.04, 0.24]	**0.164** [0.02, 0.24]	**0.272** [0.03, 0.46]
	3	**0.167** [0.06, 0.22]	**0.119** [0.02, 0.2]	**0.286** [0.13, 0.41]
	4	**0.29** [0.12, 0.38]	**0.116** [0.01, 0.3]	**0.406** [0.22, 0.58]
	5	**0.109** [0.02, 0.24]	**0.03** [0, 0.12]	**0.139** [0.05, 0.27]
	6	**0.053** [0.01, 0.15]	**0.013** [0, 0.05]	**0.067** [0.02, 0.17]
	7	**0.018** [0, 0.05]	**0.004** [0, 0.02]	**0.022** [0.01, 0.06]
Flyer	1	**-0.731** [-0.88, -0.46]	**-0.424** [-0.72, -0.1]	**-1.155** [-1.42, -0.83]
	2	**0.158** [0.02, 0.25]	**0.178** [0.05, 0.25]	**0.337** [0.13, 0.49]
	3	**0.182** [0.1, 0.23]	**0.112** [0.02, 0.2]	**0.294** [0.17, 0.4]
	4	**0.258** [0.1, 0.37]	**0.098** [0.01, 0.27]	**0.356** [0.19, 0.52]
	5	**0.083** [0.02, 0.19]	**0.023** [0, 0.08]	**0.106** [0.04, 0.21]
	6	**0.038** [0.01, 0.11]	**0.009** [0, 0.03]	**0.047** [0.02, 0.12]
	7	**0.012** [0, 0.04]	**0.003** [0, 0.01]	**0.015** [0, 0.04]
Yell	1	**-0.678** [-0.86, -0.36]	**-0.174** [-0.5, -0.01]	**-0.851** [-1.13, -0.59]
	2	**0.202** [0.08, 0.27]	**0.095** [0.01, 0.22]	**0.297** [0.11, 0.44]
	3	**0.18** [0.09, 0.23]	**0.041** [0, 0.13]	**0.221** [0.14, 0.3]
	4	**0.208** [0.06, 0.36]	**0.029** [0, 0.11]	**0.237** [0.1, 0.38]
	5	**0.056** [0.01, 0.14]	**0.006** [0, 0.02]	**0.062** [0.02, 0.15]
	6	**0.024** [0, 0.07]	**0.002** [0, 0.01]	**0.026** [0.01, 0.07]
	7	**0.007** [0, 0.02]	**0.001** [0, 0]	**0.008** [0, 0.02]
Assault	1	**-0.452** [-0.77, -0.15]	-0.013 [-0.09, 0.02]	**-0.466** [-0.77, -0.16]
	2	**0.21** [0.08, 0.28]	0.008 [-0.01, 0.05]	**0.218** [0.09, 0.29]
	3	**0.117** [0.03, 0.21]	0.003 [0, 0.02]	**0.12** [0.04, 0.21]
	4	**0.094** [0.02, 0.25]	0.002 [0, 0.01]	**0.096** [0.02, 0.25]
	5	**0.02** [0, 0.07]	0 [0, 0]	**0.021** [0, 0.07]
	6	**0.008** [0, 0.03]	0 [0, 0]	**0.008** [0, 0.03]
	7	**0.002** [0, 0.01]	0 [0, 0]	**0.002** [0, 0.01]

Supplementary References

[1] VanderWeele, T. J., Zhang, Y. & Lim, P. Brief report: Mediation analysis with an ordinal outcome. *Epidemiology* **27**, 651–655 (2016).

[2] VanderWeele, T. J. & Vansteelandt, S. Mediation analysis with multiple mediators. *Epidemiologic methods* **2**, 95–115 (2014).

[3] Imai, K., Keele, L. & Tingley, D. A general approach to causal mediation analysis. *Psychological methods* **15**, 309–334 (2010).

[4] Steen, J., Loeys, T., Moerkerke, B. & Vansteelandt, S. Flexible mediation analysis with multiple mediators. *American journal of epidemiology* **186**, 184–193 (2017).

[5] VanderWeele, T. J. Mediation analysis: A practitioner's guide. *Annual review of public health* **37**, 17–32 (2016).
