# Supplementary material for: Investigating the role of group-based morality in extreme behavioral expressions of prejudice
Source: Nat Commun. 2021 Jul 28;12:4585. doi: 10.1038/s41467-021-24786-2 (PMC8319297; doi:10.1038/s41467-021-24786-2)
Supplement: Supplementary file 3 — Reporting summary [file 41467_2021_24786_MOESM3_ESM.pdf]

nature research

Corresponding author(s): Morteza Dehghani
Last updated by author(s): Jun 3, 2021

Reporting Summary

Nature Research wishes to improve the reproducibility of the work that we publish. This form provides structure for consistency and transparency in reporting. For further information on Nature Research policies, see our Editorial Policies and the Editorial Policy Checklist.

Statistics

For all statistical analyses, confirm that the following items are present in the figure legend, table legend, main text, or Methods section.

n/a	Confirmed	
☐	☒	The exact sample size (*n*) for each experimental group/condition, given as a discrete number and unit of measurement
☐	☒	A statement on whether measurements were taken from distinct samples or whether the same sample was measured repeatedly
☐	☒	The statistical test(s) used AND whether they are one- or two-sided<br>*Only common tests should be described solely by name; describe more complex techniques in the Methods section.*
☐	☒	A description of all covariates tested
☐	☒	A description of any assumptions or corrections, such as tests of normality and adjustment for multiple comparisons
☐	☒	A full description of the statistical parameters including central tendency (e.g. means) or other basic estimates (e.g. regression coefficient) AND variation (e.g. standard deviation) or associated estimates of uncertainty (e.g. confidence intervals)
☐	☒	For null hypothesis testing, the test statistic (e.g. *F*, *t*, *r*) with confidence intervals, effect sizes, degrees of freedom and *P* value noted<br>*Give P values as exact values whenever suitable.*
☐	☒	For Bayesian analysis, information on the choice of priors and Markov chain Monte Carlo settings
☐	☒	For hierarchical and complex designs, identification of the appropriate level for tests and full reporting of outcomes
☐	☒	Estimates of effect sizes (e.g. Cohen's *d*, Pearson's *r*), indicating how they were calculated

Our web collection on statistics for biologists contains articles on many of the points above.

Software and code

Policy information about availability of computer code

Data collection	Study 1. Data from yourmorals.org, plus public data from SPLC. Study 2 & 5: Data collected via a national Qualtrics panel that was stratified with regard to age, gender, ethnicity, and political ideology. MTurk was used for studies 3 & 4.
Data analysis	Only open source software, namely R and Python, were used. All code and procedures have been made publicly available.

For manuscripts utilizing custom algorithms or software that are central to the research but not yet described in published literature, software must be made available to editors and reviewers. We strongly encourage code deposition in a community repository (e.g. GitHub). See the Nature Research guidelines for submitting code & software for further information.

Data

Policy information about availability of data

All manuscripts must include a data availability statement. This statement should provide the following information, where applicable:
- Accession codes, unique identifiers, or web links for publicly available datasets
- A list of figures that have associated raw data
- A description of any restrictions on data availability

All code and publicly available data used for this research is available at https://osf.io/67cdg/. Due to privacy restrictions, we are unable to share the Pew Religious Landscape data used for our MrsP estimation procedure or the county-level moral values estimates obtained for Study 2.

Field-specific reporting

Please select the one below that is the best fit for your research. If you are not sure, read the appropriate sections before making your selection.

☐ Life sciences ☒ Behavioural & social sciences ☐ Ecological, evolutionary & environmental sciences

For a reference copy of the document with all sections, see nature.com/documents/nr-reporting-summary-flat.pdf

Behavioural & social sciences study design

All studies must disclose on these points even when the disclosure is negative.

Study description	We report five studies that integrate spatial modeling, and experimental methods to investigate the relationship between moral values and acts of hate
Research sample	Study 2 To conduct this study, a sample of participants (N = 511) stratified by sex (51% female), age (10% to 20% per each 5-year bracket ranging from 18 to 65 or older), ethnicity (62% non-Hispanic White, 17% Hispanic, 13% Black, 7% other), and political affiliation (51% Democrat) was recruited by Qualtrics Panels. Study 3: Participants (N = 355, Mean age = 33, 54% identifying as female) were recruited from Amazon Mechanical Turk and paid $1.00. Study 4: Participants (N = 321; Mean Age = 33.92, SD = 10.88; 62% Female) were recruited via Amazon Mechanical Turk and paid $1.00 for their participation. Study 6: Participants (N = 1,049, Mean Age = 46.69, SD = 16.59; 50% Female) were sampled via a national Qualtrics panel that was stratified with regard to age, gender, ethnicity, and political ideology and randomly assigned to the control, Binding, or Individualizing condition.
Sampling strategy	Studies 2 and 5 used a stratified US based panel. Studies 4 and 5 used MTurk.
Data collection	All data collection was done online (Study 1: data collected from yourmorals.org & public SPLC data; Studies 2-5: participants recruited online
Timing	The studies were ran in a period of two years, starting in 2018.
Data exclusions	Data exclusion criteria are indicated for each study. For the behavioral studies attention checks were used in the measures.
Non-participation	Study 2: No exclusion.<br>Study 3: Three participants did not complete the MFQ and an additional 28 participants spent less than 10 seconds reading the experimental manipulation, spent less than eight minutes on the entire survey, or failed one of the MFQ manipulation checks, which were our a priori criteria to ensure data quality.<br>Study 4: Twelve participants skipped the item measuring moral wrongness and an additional 15 participants spent less than 10 seconds reading the experimental manipulation, which was our a priori cutoff to ensure data quality<br>Study 5: Twenty participants were excluded due to failing both of the MFQ attention checks.
Randomization	Study 2: No randomization.<br>Study 3: No randomization.<br>Study 4: Participants were randomly assigned to one of two conditions — a 'high moral threat' condition and a 'low moralthreat' condition — that manipulated the moral valence of a fictional outgroup.<br>Study 5: Participants were randomly assigned to the control,Binding, or Individualizing condition

Reporting for specific materials, systems and methods

We require information from authors about some types of materials, experimental systems and methods used in many studies. Here, indicate whether each material, system or method listed is relevant to your study. If you are not sure if a list item applies to your research, read the appropriate section before selecting a response.

Materials & experimental systems

n/a	Involved in the study
☒	☐ Antibodies
☒	☐ Eukaryotic cell lines
☒	☐ Palaeontology and archaeology
☒	☐ Animals and other organisms
☐	☒ Human research participants
☒	☐ Clinical data
☒	☐ Dual use research of concern

Methods

n/a	Involved in the study
☒	☐ ChIP-seq
☒	☐ Flow cytometry
☒	☐ MRI-based neuroimaging

Human research participants

Policy information about studies involving human research participants

Population characteristics	Study 2: To conduct this study, a sample of participants (N = 511) stratified by sex (51% female), age (10% to 20% per each 5-year bracket ranging from 18 to 65 or older), ethnicity (62% non-Hispanic White, 17% Hispanic, 13% Black, 7% other), and political affiliation (51% Democrat) was recruited by Qualtrics Panels. Study 3: Participants (N = 355, Mean age = 33, 54% identifying as female) were recruited from Amazon Mechanical Turk and paid $1.00. Study 5: Participants (N = 321; Mean Age = 33.92, SD = 10.88; 62% Female) were recruited via Amazon Mechanical Turk and paid $1.00 for their participation. Study 5: Participants (N = 1,049, Mean Age = 46.69, SD = 16.59; 50% Female) were sampled via a national Qualtrics panel that was stratified with regard to age, gender, ethnicity, and political ideology and randomly assigned to the control, Binding, or Individualizing condition.
Recruitment	Studies 2 and 5 used an online US based panel. Studies 3 and 4 used MTurk.
Ethics oversight	USC IRB.

Note that full information on the approval of the study protocol must also be provided in the manuscript.
